# Antioxidative, Antidiabetic, and Hypolipidemic Properties of Probiotic-Enriched Fermented Camel Milk Combined with *Salvia officinalis* Leaves Hydroalcoholic Extract in Streptozotocin-Induced Diabetes in Rats

**DOI:** 10.3390/antiox11040668

**Published:** 2022-03-30

**Authors:** Yousef M. Alharbi, Sally S. Sakr, Saleh M. Albarrak, Tariq I. Almundarij, Hassan Barakat, Mohamed F. Y. Hassan

**Affiliations:** 1 Department of Veterinary Medicine, College of Agriculture and Veterinary Medicine, Qassim University, Buraydah 51452, Saudi Arabia; yhrby@qu.edu.sa (Y.M.A.); salbarrak@qu.edu.sa (S.M.A.); tmndrj@qu.edu.sa (T.I.A.); 2 Department of Food Science and Human Nutrition, College of Agriculture and Veterinary Medicine, Qassim University, Buraydah 51452, Saudi Arabia; s.sakr@qu.edu.sa (S.S.S.); or mohamed_hassan1@agr.sohag.edu.eg (M.F.Y.H.); 3 Dairy Science Department, Faculty of Agriculture, Cairo University, Giza 12613, Egypt; 4 Food Technology Department, Faculty of Agriculture, Benha University, Moshtohor 13736, Egypt; 5 Department of Dairy Science, Faculty of Agriculture, Sohag University, Sohag 82755, Egypt

**Keywords:** *Salvia officinalis*, probiotics, fermented camel milk, antidiabetic, hypolipidemic efficiency, oxidative stress

## Abstract

Antioxidative, antidiabetic, and hypolipidemic properties of probiotic-enriched fermented camel milk (FCM) combined with *Salvia officinalis* L. leaves hydroalcoholic extract (SOHE) in streptozotocin-induced diabetes in rats were investigated. Phytochemicals analysis and antioxidant capacity indicated that *S. officinalis* contained high phenolics with super antioxidant activity. Subsequently, HPLC analysis demonstrated 13 phenolic acids and 14 flavonoids in considerable amounts with ferulic acid and resveratrol as predominant, respectively. The antidiabetic and hypolipidemic properties of FCM and SOHE were examined in a designed animal model consisting of seven treated groups for four weeks. There was a negative group (G1); the positive group (G2) received a single dose (50 mg kg^−1^) of streptozotocin (STZ) by intraperitoneal injection (i.p.); in G3, diabetic rats (DRs) orally received 5 mL FCM kg^−1^ daily; in G4, DRs orally received 50 mg GAE SOHE kg^−1^ daily; in G5, DRs orally received 5 mL FCM contains 25 mg GAE SOHE kg^−1^ daily; in G6, DRs orally received 5 mL FCM contains 50 mg GAE SOHE kg^−1^ daily; in G7, DRs orally received 50 mg metformin kg^−1^ daily. Combining FCM with SOHE at 25 or 50 mg kg^−1^ exhibited a synergistic effect in significantly lowering random blood glucose (RBG), fasting blood glucose (FBG), and improved weight gain recovery %. The hypolipidemic effect of FCM + 50 mg GAE SOHE kg^−1^ was significantly higher than using FCM or SOHE individually, and attenuation in triglycerides (TG), total cholesterol (CHO), and high- and low-density lipoproteins (HDL and LDL), and very-low-density lipoproteins (VLDL) was remarked. Combining FCM with SOHE at 25 or 50 mg kg^−1^ ameliorated liver and kidney functions better than individual uses of FCM, SOHE, or metformin. Interestingly, FCM with 50 mg SOHE kg^−1^ presented significant improvement in the activity of antioxidant enzymes, reduced glutathione (GSH), catalase (CAT), superoxide dismutase (SOD), and a substantial reduction in malonaldehyde (MDA) levels with 53.75%, 89.93%, 63.06%, and 58.69% when compared to the STZ group (G2), respectively. Histopathologically, administrating FCM + 25, 50 mg SOHE kg^−1^ or 50 mg kg^−1^ metformin showed a normal histological structure of both islets of Langerhans cells and acini. In conclusion, combining FCM with SOHE presented synergistic and therapeutical efficacy. It could be beneficial and profitable for controlling diabetes mellitus complications and protecting against oxidative stress.

## 1. Introduction

Diabetes mellitus (DM), a chronic metabolic disease, has risen to the top ten causes of death, with a 70% increase since 2000. Diabetes is also responsible for the highest increase in male deaths among the top ten, with an 80% increase since 2000 [1]. It is characterized by insulin resistance, hyperglycemia, impaired lipid and lipoprotein metabolism, oxidative stress, and subclinical inflammation [1,2]. According to statistics, one in every five adolescents aged 12–18 years and one in every four adults aged 19–34 years has prediabetes [1]. However, the number of patients diagnosed with DM is substantially increasing, which numbered 382 million in 2013 [3] and 422 million in 2014 [4]. DM predictive rates are expected to be 592 million by 2035 [5] and 642 million by 2040 [6]. Two forms of DM have been reported [7]. DM type 1 (T1DM) causes autoimmune destruction of the pancreas’ insulin-producing Langerhans β-cells. T2DM, on the other hand, is a metabolic disorder characterized by insulin resistance and relative insulin deficiency, resulting in hyperglycemia [5]. The effects of hyperglycemia can be mild to very serious for patients, mainly affecting the heart, blood vessels, eyes, neurons, nephron, and brain, which means that patients are more likely to suffer a heart attack or nephropathy neuropathy, or stroke [8]. As part of diabetes, protein, carbohydrates, and lipid metabolism are impaired. Due to the side effects of insulin and oral hyperglycemic agents, patients are becoming increasingly interested in natural products with antidiabetic properties [9].

However, a substantial increase in the global burden of diabetes is attributed to two main causes: a significant change in dietary habits resulting in obesity and overweight and physical latency [6]. Nonetheless, a healthy diet and regular physical activity can significantly reduce the risk of complications associated with diabetes with or without antidiabetic drugs [8]. Moreover, nutritional strategies are highly effective in improving metabolic control in individuals with T2DM, but there are controversies regarding which dietary composition is more efficacious [10]. In this regard, functional food ingredients and bioactive compounds are adjuncts to diet planning management and as a treatment for T2DM through glycemic control. Tiderencel et al. [11] reported a promising strategy, especially when probiotics are included [4,10,12]. Therefore, consuming functional foods may help control diabetes by regulating blood pressure, activating antioxidant enzymes, interacting with gut microbiota, and suppressing the overproduction of pro-inflammatory cytokines [13].

Generally, fermented dairy products have received extensive studies by researchers focusing on their nutritional and biofunctional properties. Its intake was associated with an inverse risk of T2DM [4,14]. In the same regard, the traditional belief in the Middle East is that regular consumption of camel milk helps prevent and control diabetes [15]. The beneficial effect of regular consumption of camel milk (CM) on diabetic patients has been empirically described for a long time. Over the years, camels’ strengths and place in human history have been guaranteed [16]. Such beneficial effects of camel milk might be due to the presence of insulin or unidentified small molecules of ‘insulin-like’ substances in the milk capable of modulating glucose levels [7,15,17]. In recent years, researchers have proven that the combination between camel milk and probiotics is more beneficial in managing T2DM in animal and human studies [4,7,11]. The fermentation of camel milk using probiotics is more effective in being more sustainable, nutritious, and health-promoting [18]. As a result, good probiotics that reduce the amount of glucose absorption in the intestines are thought to be a better way to manage hyperglycemia. The antidiabetic effect of Lactobacillus and Bifidobacterium probiotic strains has been studied in both animal and human studies [4,19]. Bifidobacteria and lactobacilli are effective in controlling hyperglycemia [4]. Most complex carbohydrates reach the colon intact if they are not digested in the small intestine. In contrast, simple sugars and disaccharides are absorbed in the small intestine after proper digestion. Bifidobacteria can utilize galacto-, manno-, and fructo-oligosaccharides at different intensities and different levels. The differences between strains of the same species, originating from various cultural sources, can be explained by their specific characteristics [4]. Moreover, modern food technologies and nutrition have suggested the involvement of probiotic cultures and prebiotic ingredients to increase some food products’ nutritional and therapeutical values [20].

Since herbs are rich sources of natural antioxidants, they are traditionally used to control and treat numerous diseases [21]. The reducing effect of many of these plants on blood glucose has been approved in animal models and clinical studies [9]. Recently, various plant-derived extracts and phytochemicals have been linked to various potentially health-promoting biological activities [22,23,24,25]. The link between a high intake of antioxidative phenolic compounds-rich foods and beverages and a lower risk of free-radical-related pathological conditions and diseases has been clearly demonstrated [26]. *Salvia officinalis* is well-known for its pharmacological properties, including anticancer, anti-inflammatory, anti-nociceptive, antioxidant, antimicrobial, antimutagenic, antidementia, hypoglycemic, and hypolipidemic effects [27], and most of its active components have been identified. It has been established that the antioxidant effects are mainly due to the phenolic compounds of the plant [21,28].

Recently, many in vitro and in vivo studies have been available on the health benefits of probiotic fermented camel milk (FCM). Still, few mainly focused on the antidiabetic potential of probiotic camel milk or *S. officinalis* separately. To the best of our knowledge, the antioxidative and antidiabetic potential of FCM combined with *S. officinalis* hydroalcoholic extract (SOHE) in the form of functional beverages was not studied yet. Indeed, combining FCM with *S. officinalis* extract to formulate an innovative drink is an excellent idea to attain a natural product with superior protective and therapeutical properties, as hypothesized in the present work. Therefore, the producing potential of FCM incorporated SOHE to attain a functional and therapeutical drink was targeted. Subsequently, the evaluation of the antioxidative, antidiabetic, and hypolipidemic properties of FCM combined with SOHE was investigated through in vivo animal model.

## 2. Materials and Methods

### 2.1. Preparation of FCM

Camel milk was obtained from the College of Agriculture and Veterinary Medicine Farm at Qassim University in Saudi Arabia (SA) between February and March 2021. The samples were immediately transported to the laboratory under cold conditions. The milk sample was heated at 85 °C for 15 min before being cooled to the inoculation temperature (42 °C), [29,30]. The ABT-5 starter consisting of *Streptococcus thermophiles*, *Lactobacillus acidophilus*, and *Bifidobacterium bifidum* in freeze-dried direct-to-vat set form (DVS), was obtained from Chr. Hansen in Copenhagen, Denmark. The ABT-5 starter was 1 g per 1 L of camel milk. After that, samples were incubated at 42 °C for 4–5 h to achieve a pH of 4.6–4.7 before being cooled for 12 to 18 h. Aseptically, samples (50 mL) were collected in sterile bags for microbiological analysis (only to check the viable bacterial count, data not presented).

### 2.2. Preparation of S. officinalis Leaves Hydroalcoholic Extract

*S. officinalis* leaves were purchased from El Resieny market, Buraydah city, KSA. The authentication of the plant was carried out by plant expertise (Dr. Mokded Rabhi), College of Agriculture and Veterinary Medicine, Qassim University, KSA. The dried leaves were mechanically powdered then kept in opaque polyethylene bags until used under 4 ± 1 °C. Approximately 500 g of *S. officinalis* were extracted with 2500 mL ethanol (50%) 3 times to prepare *S. officinalis* hydroalcoholic extract (SOHE). The filtered extract was concentrated by a rotary evaporator (Büchi, Flawil, Switzerland) at 40 °C to evaporate the remaining solvent, frozen overnight, then freeze-dried for 96 h at −52 °C using (CHRIST, Alpha 1–2 LD plus, Martin Christ Gefriertrocknungsanlagen GmbH, Osterode, Germany), and 0.032 mbar [31]. Freeze-dried samples were pulverized using a porcelain morsel to prepare homogeneous powder that was kept in the dark packages at 4 ± 1 °C until used.

### 2.3. Determination of Total Phenolic Content (TPC), Total Carotenoids (TC), Total Flavonoids (TF), and Total Flavonols (TFL) in S. officinalis Leaves

The TPC of *S. officinalis* leaves was determined using Folin–Ciocalteu reagent, according to Yawadio Nsimba et al. [32]. Briefly, an appropriate sample was extracted with 70% methanol. Aliquots of clear supernatant were mixed with (1:10) diluted Folin–Ciocalteu reagent for 5 min, then Na₂CO₃ (7.5%) was added to stop the reaction. After 60 min, the optical density (OD) was measured and compared to the standard curve of gallic acid (GA) solution (R2 = 0.99). TPC content was expressed as milligrams of gallic acid equivalents (GAE) per 100 g (mg of GAE g^−1^ dw). For TC determination, 1 g of the freeze-dried sample was repeatedly extracted with a mixture of acetone and petroleum ether (1:1, *v*/*v*), according to Yuan et al. [33]. The upper phase was collected, washed several times with water, and combined with crude extracts. The petroleum ether will be added to the solution to prepare a known volume. TC content has been spectrophotometrically determined at 451 nm then expressed as mg 100 g^−1^ dw. The TF content of *S. officinalis* leaves was determined in the methanolic extract. Aliquots of clear extract were mixed with AlCl_3_ (2%), kept in the dark for 60 min, and then OD was measured at 420 nm. The TFL content of *S. officinalis* leaves was determined by mixing aliquots of methanolic extracts with sodium acetate (5%). After 5 min, AlCl_3_ (2%) was added, kept in the dark for 150 min, and then OD was measured at 440 nm according to Mohdaly et al. [34]. The content of TF and TFL were expressed as mg quercetin equivalent (QE) per g^−1^ (mg QE g^−1^).

### 2.4. Antioxidant Capacity Determination

Radical scavenging activity was measured spectrophotometrically based on the bleaching of DPPH radicals purple solution according to Yawadio Nsimba et al. [32]. The DPPH radical scavenging activity percentage was used to plot the Trolox calibration curve. The antiradical activity was expressed as micromoles of Trolox Equivalents (TE) per gram (µmol TE g^−1^). The radical scavenging activity (RSA) of *S. officinalis* leaves against the stable ABTS (2,2′-azino-bis(3-ethylbenzothiazoline-6-sulphonic acid)) radical cation was measured using the method of Barakat and Rohn [35]. A Trolox calibration curve was plotted as a function of the ABTS radical cation scavenging activity percentage. The final results were expressed as micromoles of Trolox Equivalents (TE) per gram (µmol of TE g^−1^). The antioxidant percentage of *S. officinalis* leaves was assessed in terms of β-carotene bleaching compared to butylated hydroxyanisole (BHA) according to Koleva et al. [36]; the results were given as a BHA-related percentage. The chelating activity of *S. officinalis* leaves was measured as protocoled by Zhao et al. [37]. The inhibition % of ferrozine-Fe^2+^ complex creation as metal chelating action was calculated and presented as (mg mL^−1^) when ethylenediaminetetraacetic acid (EDTA) as a positive control was used.

### 2.5. Quantification of Phenolic Compounds in S. officinalis Leaves by HPLC-DAD

The phenolic compounds in *S. officinalis* leaves were determined by the HPLC system HP1100 (Agilent Technologies, Palo Alto, CA, USA) equipped with an autosampler, quaternary pump, and diode array detector DAD (Hewlett Packard 1050), using a column (Altima C18, 5 × 150 mm, 4.6 mm ID) and a guard column Altima C18, 5 mm (Alltech) according to Kim et al. [38]. The solvent system contained a gradient of A (Acetic acid 2.5%), B (Acetic acid 8%), and C (Acetonitrile). The 10 µL of solvent was injected at a flow rate of 1 mL min^−1,^ and separation was performed at 25 °C. The peaks of phenolic compounds (µg g^−1^) were identified by comparing the results with library and external standards’ retention times and mass spectrums. The external standards such as naringin, hispidulin, cirsimaritin, luteolin, chrysin, and resveratrol were purchased from Sigma-Aldrich, St. Louis, MI, USA.

### 2.6. Preparation of FCM Incorporated SOHE

Prepared FCM was freshly mixed with freeze-dried SOHE to prepare FCM containing 25 or 50 mg GAE of SOHE per 100 mL camel milk directly before oral administration of rats.

### 2.7. Animals and Experimental Design

This study used Wistar rats (56 adult males) weighing between 150 and 175 g. All experiments were approved by the Institutional Animal Ethics Committee (IAEC) of QU, KSA, which is governed by the Control and Supervision of Experiments on Animals (CPCSEA) Committee of the National Committee of BioEthics (NCBE), which implements regulations related to the ethics of research on living creatures. Under standard laboratory conditions, the animals were housed in air-conditioned polypropylene cages and kept at 24 ± 1 °C under standard laboratory conditions. After ten days of acclimatization, rats were randomly divided into 7 groups (8 rats/group) and housed in new cages under controlled conditions of 24 ± 1 °C, 40–45% relative humidity, and a 12 h light/dark cycle. The rats were labeled, their body weight (BW) was recorded, and their random blood glucose (RBG) was measured with a Glucometer (Accu-Check, Roche, Mannheim, Germany). The rats were fed a commercial standard pellet diet and were given water *ad libitum* [39]. The different groups of rats were treated as follows: Group 1 (normal rats, NR) received an intraperitoneal injection of saline solution and 5 mL distilled water orally per day. For the induction of diabetes mellitus in experimental rats, the animals fasted overnight. All rats, except the NR group, were administered a single intraperitoneal injection of freshly prepared solution of STZ (Sigma-Aldrich, Merck, St. Louis, MI, USA) in 0.1 M citrate buffer (pH = 4.5) at the dose of 50 mg kg^−1^. Diabetes was assessed in rats by monitoring the fasting blood glucose (FBG) level 48 h after the injection of STZ using a Glucometer (Accu-Check, Roche, Germany). Experimental rats with an FBG > 200 mg dL^−1^ were considered diabetic and were used in the study. Animals were randomized based on their body mass and RBG and divided into six groups: Group 2, diabetic rats (DR) were administered 5 mL distilled water orally per day, Group 3 (DR + FCM) diabetic rats orally administered 5 mL FCM kg^−1^ daily, Group 4 (DR + SOHE) rats orally administered 50 mg GAE SOHE kg^−1^ daily, Group 5 (DR + FCM-SOHE1) rats orally administered 5 mL FCM contains 25 mg GAE SOHE kg^−1^ daily, Group 6 (DR + FCM-SOHE2) rats orally administered 5 mL FCM contains 50 mg GAE SOHE kg^−1^ daily, Group 7 (DR + Metf) rats orally administered 50 mg standard drug metformin kg^−1^ daily. Metformin is the first-line medication for the treatment of type 2 diabetes. Hence, metformin was chosen as the reference drug in this study [40].

At the end of 28 days, animals fasted for 12 h with free access to water. On the 29^th^ day, rats were anesthetized with a mixture of alcohol, chloroform, and ether (1:2:3) according to Leila et al. [41] with minor modification as the experimental period was extended one week more to ensure the effect. Blood samples were collected from the heart puncture of all the animals. Blood tubes were subjected to serum separation by centrifugation at 4000× *g* for 30 min under cooling to attain serum used for various biochemical parameters. The biochemical parameters were determined using suitable kits and a blood chemistry analyzer (HumaLyzer 4000, Human Gesellschaft für Biochemica und Diagnostica mbH Wiesbaden, Germany). Animals were sacrificed, and rats were dissected to collect the pancreas, twice washed with saline solution and fixed in 10% Formaldehyde according to Zafar and Naqvi [42].

#### 2.7.1. Determination of Fasting Blood Glucose Level (FBG), Lipid Profile, Liver and Kidneys’ Functions

FBG (mg dL^−1^) was determined using an enzymatic colorimetric test kit applying the GOD-PAP method. Lipid profile including triglycerides (TG, mg dL^−1^), total cholesterol (CHO, mg dL^−1^) using enzymatic colorimetric test kit applying GPO-PAP method, high-density lipoproteins (HDL, mg dL^−1^) using enzymatic colorimetric direct homogenous test kit following company protocols were determined. Low-density lipoproteins (LDL, mg dL^−1^) and very-low-density lipoproteins (VLDL, mg dL^−1^) were mathematically calculated according to Friedewald et al. [43]. Liver functions such as alanine aminotransferase (ALT, UL^−1^), aspartate aminotransferase (AST, UL^−1^), alkaline phosphatase (ALP, UL^−1^), total bilirubin (T. Bili, mg dL^−1^), and direct bilirubin (D. Bili, mg dL^−1^) in blood serum using alanine aminotransferase kit (EC 2.6.1.2), aspartate aminotransferase kit (EC 2.6.1.1), optimum alkaline kit (EC 3.1.3.1) and photometric test kits for total and direct bilirubin were examined, respectively. Kidney functions such as total protein (T. protein, g dL^−1^), albumin (g dL^−1^), creatinine (mg dL^−1^), and urea (mg dL^−1^) concentrations using photometric, colorimetric test kits applying Biuret method, photometric, colorimetric test kits applying BCG method, photometric, colorimetric test kits, fully enzymatic test kit applying GLDH method were respectively determined according to the instructions of the manufacturer. Globulin (g dL^−1^) was calculated by subtracting albumin from T. protein concentrations. Blood urea nitrogen (BUN, mg dL^−1^) was calculated by multiplying urea concentration by 0.47. All biochemical examination kits were purchased from Human Co., Wiesbaden, Germany. The atherogenic index (AI) was calculated according to Nwagha et al. [44].

#### 2.7.2. Oxidative Stress Biomarkers

Reduced glutathione (GSH, µg dL^−1^) was estimated using GSH colorimetric assay kit (E-BC-K030-S, Elabscience, Houston, TX, USA) according to the method described by Beutler et al. [45]. Lipid peroxidation was estimated using a malondialdehyde (MDA, nmol mL^−1^) colorimetric assay kit (E-BC-K025-S, Elabscience, Houston, TX, USA) by measuring thiobarbituric acid reactive substance (TBARS) and expressed in terms of MDA content according to Ohkawa et al. [46]. MDA, an end product of fatty acid peroxidation, forms a colored complex reacting with Thiobarbituric acid (TBA). The absorbance of the supernatant was measured at 532 nm, and the results were calculated as nmol mL^−1^. Superoxide dismutase (SOD, U L^−1^) activity using SOD typed activity assay kit (E-BC-K022-S, Elabscience, Houston, TX, USA) was determined according to Giannopolitis and Ries [47]. The color reaction was measured at 550 nm, expressed as U L^−1^. Catalase (CAT, U L^−1^) activity was determined using a CAT activity assay kit (E-BC-K031-S, Elabscience, Houston, TX, USA) according to the method of Aebi [48]. All Oxidative stress markers were determined using a blood chemistry analyzer (HumaLyzer 4000, Human Gesellschaft für Biochemica und Diagnostica mbH, Wiesbaden, Germany).

#### 2.7.3. Histopathological Examination

Autopsy of the fixed pancreas in 10% formal saline up to 48 h for different experimental groups were taken. For dehydration, samples were washed in water, then serial dilutions of alcohols were prepared. At 56 °C in a hot air oven, cleared in xylene and embedded in paraffin for 24 h. After microtome sectioning, the tissue sections were deparaffinized and immediately stained with hematoxylin-eosin (H&E). The stained sections were diagnosed for histopathological alterations in pancreas architecture, and their photomicrographs were taken according to Banchroft et al. [49]. Subsequently, the results of undefined experimental groups were re-diagnosed by two pathologists to confirm the result observation.

### 2.8. Statistical Analysis

Statistical analysis was performed using SPSS (Ver. 22.0 for Windows, IBM, Houston, TX, USA). Experimental results were expressed as mean ± SE. Statistical significance was tested with one-way ANOVA followed by a post hoc test, and *p*-values < 0.05 were applied according to Steel et al. [50].

## 3. Results

### 3.1. Phytochemicals and Antioxidant Capacity of S. officinalis Leaves

The quantitative analysis of *S. officinalis* L. phytochemicals and related antioxidant activities was performed using DPPH and ABTS radical scavenging, β -carotene–linoleic acid bleaching activities, and chelating ability (CA). TPC content was 102.81 mg GAE g^−1^ as shown in Table 1. The TC content was 4.11 µg g^−1^. The TF and TFL contents were mg QE g^−1^, respectively. Furthermore, DPPH-RSA and ABTS-RSA were used to track the progression of antioxidant activities. The results showed 337.62 mol of TE g^−1^ for DPPH-RSA and 374.62 mol of TE g^−1^ for ABTS-RSA, respectively. The inhibition percentage of linoleic acid radicals was calculated as 63.27% compared to BHA using the β-Carotene bleaching (β-CB) assay. Furthermore, evaluation of the metal-chelating activity revealed 71.21 mg g^−1^, which seems to be proficient in interfering with Fe^2+^–ferrozine complex formation, indicating its capability to chelate oxidation metals.

### 3.2. Quantification of Phenolic Compounds in S. officinalis Leaves Extract

The quantitative analysis of phenolics in *S. officinalis* leaves extract was carried out, and data are tabulated in Table 2. Thirteen separated phenolic acids and fourteen flavonoids were identified in detectable amounts in SOHE. The most abundant hydroxycinnamic acids were ferulic acid (814.17 mg 100 g^−1^), followed by caffeic acid (39.15 mg 100 g^−1^), cinnamic acid (28.34 mg 100 g^−1^), rosmarinic acid (13.35 mg 100 g^−1^), *p*-coumaric acid (12.27 mg 100 g^−1^), *O*-coumaric acid (7.08 mg 100 g^−1^), and chlorogenic acid (1.27 mg 100 g^−1^). For hydroxybenzoic acids, benzoic acid (89.37 mg 100 g^−1^) was detected as the major phenolic acid, followed by vanillic acid (49.73 mg 100 g^−1^), *p*-hydroxybenzoic acid (23.28 mg 100 g^−1^), ellagic acid (5.57 mg 100 g^−1^), syringic acid (5.09 mg 100 g^−1^), and gallic acid (1.083 mg 100 g^−1^). The SOHE is rich in flavonoids content, as shown in Table 2. Flavonoids such as resveratrol (1876.95 mg 100 g^−1^) and kaempferol (356.52 mg 100 g^−1^) were detected in higher amounts, followed by chrysin (102.57 mg 100 g^−1^), epicatechin (98.12 mg 100 g^−1^), apigenin (97.17 mg 100 g^−1^), quercetin (91.07 mg 100 g^−1^), cirsimaritin (89.43 mg 100 g^−1^), luteolin (87.12 mg 100 g^−1^), and luteolin-7-O-glucoside (25.18 mg 100 g^−1^). Rutin, myricetin, naringin, and hispidulin were detected in moderate amounts, while catechin was detected in low content. It is shown that flavonoids exhibited superior amounts in SOHE.

### 3.3. The Hypoglycemic Efficiency and Weight Gain %

The hypoglycemic efficiency and weight gain % of SOHE, FCM, FCM + SOHE at 25 and 50 mg kg^−1^ and metformin at 50 mg kg^−1^ on STZ-induced diabetes in rats were monitored; data are tabulated in Table 3. STZ injection affected the rats’ weight directly during the first week, then very low weight gain % was recorded on week-2 and week-4. The best efficient treatment in recovering rats’ weight was administrating FCM + SOEH followed by FCM or metformin on week-2 and week-4. SOEH alone was recorded as the lowest weight gain enhancer on week-2 and week-4 when compared with normal rats. After two weeks, a slight attenuation has been remarked in FCM and SOHE groups, but a significant improvement has been remarked with combined FMC with 25 or 50 mg kg^−1^. After week-4, FCM + SOHE at 50 mg kg^−1^ exhibited a powerful efficacy in reducing RBG better than metformin at 50 mg kg^−1^, as shown in Table 3. The efficiency of SOHE in reducing RBG was better than FCM. However, combining FCM with SOHE at 25 or 50 mg kg^−1^ exhibited a synergistic effect in lowering RBG.

FBG measurement confirmed that FCM with SOHE impressively attenuated FBC closely to normal rats. Interestingly, combining FCM with 25 or 50 mg kg^−1^ SOHE attenuated the glucose level in blood serum and significantly improved its level compared with normal or metformin groups.

### 3.4. The Hypolipidemic Efficiency

The hypolipidemic efficiency of SOHE, FCM, FCM + SOHE at 25 and 50 mg kg^−1^ and metformin at 50 mg kg^−1^ on Streptozotocin-induced diabetes in rats were determined; results are illustrated in Table 4. A significant increase in TG, CHO, LDL, and VLDL levels of diabetic rats was noted. However, a significant decrease in HDL levels was recorded with STZ injection compared to normal rats (G1). Administration of FCM or SOHE individually were moderately improved the lipid profile, whereas a combination exuded more effect than using them separately. The rats’ treatments with SOHE, FCM, and FCM + SOHE at 25 or 50 mg kg^−1^ significantly attenuated the TG, CHO, LDL-CHO, and VLDL-CHO levels compared with normal and metformin groups. SOHE, FCM, and FCM + SOHE at 25 or 50 mg kg^−1^ treatments significantly increased the HDL-CHO and decreased VLDL-CHO levels. The most efficient treatment for improving the blood profile was FCM with 50 mg SOHE kg^−1^. Interestingly, the rate of HDL-CHO increase was recorded as 19.99%, 33.33%, 39.98%, and 69.98%, whereas an LDL-CHO decrease was noted as 45.07%, 57.17%, 56.96%, and 72.51% after SOHE, FCM, and FCM + SOHE at 25 or 50 mg kg^−1^ treatments, respectively. The VLDL-CHO level was improved associatively with treatments in a type and dose-dependent manner. FCM with 50 mg SOHE kg^−1^ was the best treatment, whereas it reduced the VLDL by more than 50% compared with the STZ group (G2). Interestingly, the AI was significantly increased after STZ injection (G2) compared with normal rats (G1). The most efficient treatments in attenuating the atherogenicity complication were FCM + SOHE at 25 or 50 mg kg^−1^, which present a superior effect better than FCM or SOHE individually or even using metformin.

### 3.5. The Liver’s Functions

STZ injection substantially raised serum ALT, AST, and ALP enzyme levels in G2 rats as diabetes complications compared to normal rats (GI). T. Bili and D. Bili levels were significantly increased in STZ-treated rats (Table 5). Administration of FCM or SOHE individually improved the liver’s function, whereas combination exhibited an accumulative effect than using them in separate forms. SOHE was better than FCM to improve liver functions. Interestingly, FCM + SOHE at 25 or 50 mg kg^−1^ treatments substantially reduced the alterations in liver functions caused by STZ injection to be close to normal values in GI (Table 5). However, combining SOHE with FCM was much better than using them separately. FCM + SOHE at 25 or 50 mg kg^−1^ markedly improved the liver enzymes (as presented in ALT, AST, ALP) and some liver functions such as T. Bili and D. Bili in a type and dose-dependent manner even better than using metformin.

### 3.6. The Kidneys’ Functions

The nephroprotective efficiency of SOHE, FCM, FCM + SOHE at 25 and 50 mg kg^−1^ and metformin at 50 mg kg^−1^ on streptozotocin-induced diabetes in rats were investigated; results are illustrated in Table 6. STZ injection substantially raised serum creatinine, urea, and BUN levels in G2 rats compared to normal rats (GI). Conversely, T. protein, albumin, and globulin levels were significantly decreased in STZ-treated rats (Table 6). SOHE, FCM, and FCM + SOHE at 25 or 50 mg kg^−1^ treatments substantially attenuated the alterations in creatinine, urea, and BUN caused by diabetes complications. At the same time, they increased T. protein, albumin, and globulin levels to be close to normal values in GI (Table 6). The most efficient improvement was markedly recorded with FCM with 50 mg SOHE kg^−1^ even better than using metformin when compared to normal rats (G1).

### 3.7. Antioxidant Biomarkers

As shown in Table 7, injection of STZ significantly reduced GSH, CAT, and SOD enzymes levels and increased the MDA level in blood serum of DR (G2) compared to normal rats (G1). Treated rats with SOHE, FCM, FCM + SOHE at 25 and 50 mg kg^−1^ and metformin at 50 mg kg^−1^ presented significant improvement in the activity of antioxidant enzymes GSH, CAT, and SOD as well as a substantial reduction in MDA levels (Table 7). The best treatment was FCM with 50 mg SOHE kg^−1^, which recorded an improvement rate of 53.75%, 58.69%, 89.93%, and 63.06% for GSH, DMA, CAT, and SOD when compared to the STZ group (G2), respectively. However, administration of SOHE and FCM + SOHE at 25 or 50 mg kg^−1^ exuded a synergistic effect in attenuating antioxidant levels and combating the autoxidation process resulting in low MDA levels even better than GI and G7. Similarly, both FCM with 25 mg SOHE or 50 mg SOHE kg^−1^ enhanced the enzymatic defense system significantly compared to normal rats (G1).

### 3.8. Effects of Probiotic-Enriched FCM Combined with S. officinalis Pancreas Histoarchitecture

**Figure 1:**
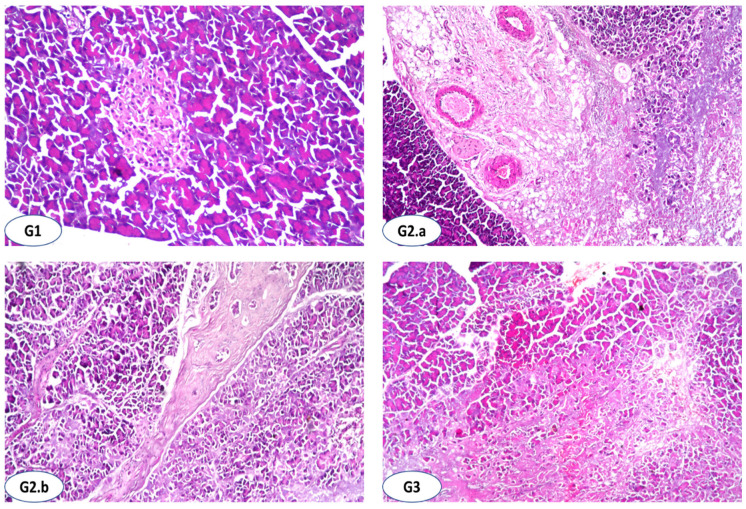

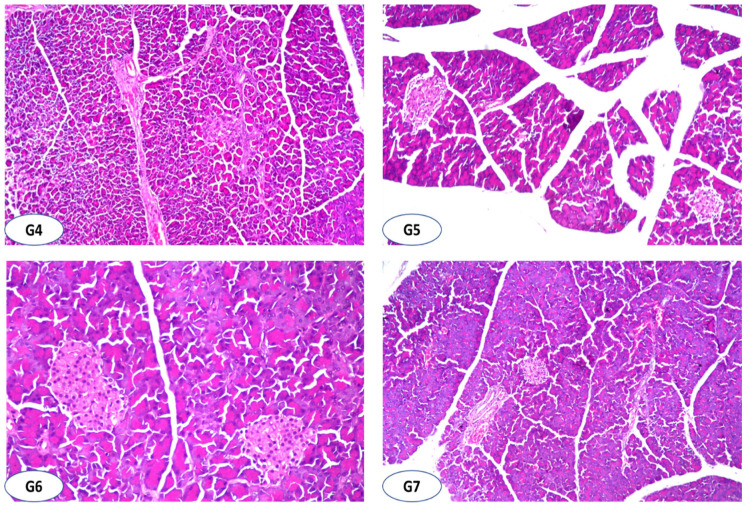
Histopathological findings of rats’ pancreas of the experimental groups (Hematoxylin-Eosin, HE; ×16). (**G1**): Showing normal histological structure of the islets of Langerhans cells as endocrine portion with the acini and duct system of the exocrine (×16). (**G2**) (a,b): Showing interlobular fibrosis with atrophy of islets of Langerhans and the acini. (**G3**): Showing intact islet of Langerhans cells with fine fibrosis between the lobules. (**G4**): Showing atrophy in islets of Langerhans cells with necrobiosis in the acini. (**G5**–**G7**): Showing normal histological structure of both islet of Langerhans cells and acini.

The results of the biochemical investigations were supported by histopathological examination. Table 8 and Figure 1 show the degree of histological changes in the underlying structure of the rat’s pancreas in various experimental groups treated with SOHE (G3), FCM (G4), FCM + SOHE at 25 or 50 mg kg^−1^ (G5 and G6), and metformin at 50 mg kg^−1^ (G7). In the current investigation, no histopathological alteration and normal histological structure of the islets of Langerhans cells as the endocrine portion and the acini and ducts as the exocrine portion were recorded in the pancreas of the pancreas control group (Figure 1G1). The histoarchitecture of the STZ-treated rats (G2) showed Fibroblastic cell proliferation was detected between the lobules with atrophy of the islet of Langerhans cells (Figure 1G2(a,b)). Subsequently, severe (+++) atrophy of islets of Langerhans and interlobular fibrosis as well as moderate (+++) necrobiosis in acini were diagnosed. In G3, the islet of Langerhans was histologically intact normal, associated with mild focal fibroblastic cells proliferation in between the lobules and mild (+) atrophy in islets of Langerhans (Figure 1G3). Administration of SOHE shows substantial attenuation in histopathological alteration resulting in mild (+) necrobiosis in acini and interlobular fibrosis had been observed (Figure 1G4). Combining FCM with SOHE at 25 mg kg^−1^ presented mild (+) atrophy in the islet of Langerhans cells associated with necrobiosis in the acini (Figure 1G5). In comparison, FCM with SOHE at 50 mg kg^−1^ and metformin as a drug dose at 50 mg kg^−1^ demonstrated no histopathological alteration in both islets of Langerhans cells and the acini (Figure 1G6,G7).

## 4. Discussion

Consuming functional foods have been proven to control diabetes by regulating blood pressure, activating antioxidant enzymes, interacting with gut microbiota, and suppressing the overproduction of pro-inflammatory cytokines [13]. A promising strategy, especially when probiotics are included, has been reported [4,10,12]. With the low acceptable organoleptic characteristics of CM and FCM, adding *S. officinalis* extract is an excellent idea to enhance its protective and therapeutical properties, as innovatively hypothesized in the current work. Phytochemicals included in this herb are supposed to be effective free radical scavengers and are considered plant-based superior antioxidant agents [21,24,25,26,51]. The antidiabetic and antioxidative stress efficiency of *S. officinalis* was approved in recent studies [9,52]. Interestingly, *S. officinalis* has been successfully incorporated in fermented milk [21,53], but no studies have yet been established about its incorporation in FCM.

The valuable phytochemical content and antioxidant activities of *S. officinalis* were higher than those reported by Roby et al. [54] and corresponded with those reported by Murat et al. [55]. Biologically active components, such as phenolic chemicals exhibit antioxidant activity by breaking down lipid oxidation chain reactions and supplying hydrogen to active free radicals. The phenolic hydroxyl groups were responsible for phenolics’ ability to scavenge radicals and inhibit them [26,55]. This phenolic acid has been reported as an efficient antioxidant component that inhibits the formation of hydrogen peroxide, hydroxyl radicals, and superoxide anion [51,54]. A direct association exists between increased phenolic component concentration and antioxidant capacity [56]. The metal chelating activity of *S. officinalis* appears to be capable of interfering with the formation of the “Fe2+–ferrozine” complex, implying that it can capture “ferrous” ions before “ferrozine.”

Biologically active components, such as phenolic compounds, have been described as practical antioxidant components, including hydrogen peroxide, hydroxyl radical, and superoxide anion, and were effective against numerous metabolic diseases [12,57]. Quantifying phenolics in *S. officinalis* leaves indicated considerable numbers of phenolic acids and Flavonoids. The identified phenolics number was higher than the number of identified compounds in *S. officinalis* by Roby et al. [54] but agreed that they identified 12 components with ferulic acid as predominant phenolic acid. Interestingly, current research noticed a valuable amount of identified flavonoids higher than confirmed previously by Roby et al. [54] and Walch et al. [58]. The results reflect that consuming *S. officinalis* could present a phenolics-rich drink in both polar and nonpolar forms, which consider a good source of natural antioxidants with potential health benefits [40,57,59].

On the other hand, prolonged hyperglycemia is a primary cause of most complications of diabetes. Indeed, chronic hyperglycemia is thought to lead to metabolic impairments and oxidative stress in diabetes [1,28,41]. Our recent in vivo study indicated that *S. officinalis* and FCM reported significant decreases in RBG and FBG in experimental rats in a dose and type-dependent manner, as similarly shown [5,8,52,60]. These findings support the results of our study, which confirms that *S. officinalis* and FCM possess hypoglycemic effects. FCM + SOHE at 25 and 50 mg kg^−1^ strongly reduced RBG and FBG compared to SOHE and FCM individually because they could combine polyphenols as effective antioxidants and insulin-like substances from camel milk which are capable of modulating glucose levels [7,15,17]. Practically, the administration of FCM and SOHE individually or in combination was most helpful in body weight recovery in a dose and type-dependent manner [57]. Therefore, the restoration of cognitive function observed in the diabetic animals in this study may be partly due to the ability of SOHE to attenuate hyperglycemia.

Elevated serum triglycerides and cholesterol levels in the STZ-diabetes rats indicate impaired fat metabolism due to diabetes complications [60]. Administration of SOHE, FCM, FCM + SOHE at 25 and 50 mg kg^−1^ were significantly attenuated the drastic changes in lipid profile when used separately, as similarly indicated [7,60]. A combination of FCM with SOHE at 25 or 50 mg kg^−1^ efficiently improved TG, CHO, LDL, and VLDL levels, indicating an accumulative or synergistic effect. This might be due to phenols, antioxidants, and carotenoids [27,58,59]. Several clinical studies have indicated that FCM consumption can lower cholesterol and improve lipid profile which might help control insulin levels and attenuate diabetes complications [61]. Shori et al. [8] reviewed that camel milk has a powerful effect in reducing blood glucose levels and insulin requirements. It limits diabetic complications such as elevated cholesterol levels, liver and kidney diseases, decreased oxidative stress, and delayed wound healing. Furthermore, the fermentation of camel milk in the presence of probiotic bacteria could increase the potential therapy of camel milk to control diabetes [8]. The combination of SOHE with FCM presented therapeutical benefits and could enhance multi responses to help recover and attenuate diabetes complications.

Significant changes were documented in the liver diagnostic markers in treated groups compared to the negative control group (G1) (Table 5). The significant increase in enzymes activities of ALT, AST, ALP, and T. Bili and D. Bili in G2 due to STZ administration was previously noted [62] as a normal deterioration related to liver injury in DM [6]. Although those increments significantly declined in Groups Two, Three, and Seven as a result of FCM, SOHE, and metformin, respectively, they were more significantly decreased in G5 and G6 as a result of the SOHE and FCM combination with no significant differences in the SOHE amount. The high content of phenolic acids can conclude this and flavonoid compounds in the *S. officinalis* extract, previously described as an anti-inflammatory and non-toxic substance for the liver [27,63,64]. Moreover, the bioactive peptides derived from the action of probiotic strains on camel milk proteins during fermentation enhanced this effect [65]. Additionally, probiotic strains presented in the final product may contribute to this improvement. A recent study on the Protective role of Probiotic supplements in hepatic steatosis [66] showed that the probiotic strain mix plays a vital role in preventing and treating metabolic disorders, improving lipid profiles, enhancing liver function markers, and suppressing inflammatory inflammation marker levels.

As the rats injected with STZ (G2) had a highly significant increase in blood glucose levels (Table 3), the kidney functions of rats in the same group were also worsened (Table 6). This relationship is highly interconnected because the higher blood glucose levels increase, the more kidneys’ filtering units are damaged, leading to kidney failure [67]. Hence, DM became one of the leading causes of end-stage kidney disease (ESKD), known as Diabetic nephropathy [68]. Data presented in Table 6 clearly showed the recovery in all kidney functions of diabetic rats’ oral administered with FCM (G3), SOHE (G4), or their combination (G5 and G6). The increased levels of T. protein, albumin, and globulin and the decrease in creatinine, urea, and BUN were highly significant in G6 compared to all groups, even the negative control group (G1) and (G7) with metformin administration. In a previous study on the effect of camel milk on kidney function of diabetic rats [8]. An enhancement of kidney functions parameters to the normal level in diabetic rats fed camel milk was shown. Regarding the positive effect of *S. officinalis* extract on the recovery of the kidneys’ function, it was previously explained that carnosic acid, rosemarinic acid, caffeic acid, and essential oil are responsible for protecting the body against oxidative stress and free radical attack. On the other hand, it was suggested that this extract has hypoglycemic effects in normal and diabetic animals, reducing liver glucose production and raising the action of insulin correlated with improving kidney functions [63,67].

In the current study, STZ administration markedly decreased GSH, SOD, and CAT and increased MDA levels in the serum of diabetic rats compared to normal rats as well documented [28]. GSH is a non-enzymatic antioxidant that is found in all mammalian cells. With its oxidized form, GSSG, GSH acts as a cofactor for numerous detoxifying enzymes (GPx, GST, and others) against oxidative stress and maintains cellular redox balance [69]. In the same context, SOD catalyzes the dismutation of two molecules of superoxide anion (^•^O_2_) to hydrogen peroxide (H_2_O_2_) and molecular oxygen (O_2_), consequently rendering the potentially harmful superoxide anion less hazardous [70]. MDA is the first lipid peroxidation product and is one of the important markers of oxidative stress. ROS increases the risk of tissue damage and causes lipid peroxidation as determined by the catabolite malondialdehyde marker [71]. Administrating SOHE, FCM, FCM + SOHE at 25 and 50 mg kg^−1^ and metformin at 50 mg kg^−1^ ameliorated the diverse effects of STZ by restoring the altered activity of antioxidant agents such as SOD, CAT, and GSH and may deactivate the process of producing the MDA [57]. The combination of FCM with SOHE exhibited superior efficiency in antioxidation prevention better than metformin, as previously approved that *S. officinalis* has a metformin-like effect [40]. Based on the present study’s findings, oral administration of SOHE decreased lipid peroxidation and increased the antioxidant enzymes SOD and CAT levels in STZ-diabetic rats [57]; the efficiency was markedly increased when FCM was combined, presenting small molecules of ‘insulin-like’ substances in the milk capable of modulating diabetes and attenuating its complications [7,15,17]. SOHE, FCM, FCM + SOHE at 25 and 50 mg kg^−1^ diminished the increase in MDA levels and restored total antioxidant power in the STZ-treated rats. These protective effects may be due to the potent antioxidative activity of *S. officinalis* and FCM in abundant polyphenols, which efficiently reduces complications related to oxidative stress [40,54,58].

As appeared in the pancreas histoarchitectures under the current study, photomicrographs of the G1 section show normal histological structure. The decrease in size and irregular borders of normally developed islets of Langerhans in G2 (a and b) compared to G1 have markedly appeared. As a positive control among study groups, the STZ-treated rats (50 mg kg/d/ip) images of the pancreas show derangement of Langerhansߣ islets, lack of compaction of glandular cells, and interlobular fibrosis. Thus, STZ seemed extremely toxic to the pancreatic cells, thereby severely damaging the β-cells [72]. Earlier studies on laboratory animals have demonstrated that STZ extensively reduces β-cells mass and destroys pancreatic islet volume [73]. Moreover, Mohamad et al. [74] explained that it was clear that pancreatic cells of diabetic rats showed extensive damage and loss of architecture with marked atrophy of the islets of Langerhans accompanied by a reduction in the number and size of β-cells. As seen in the G2.a micrograph, the islets of Langerhans showed necrosis, and the islet cells were attracted by the immune system next to a blood visile degeneration of Langerhans. The same finding was previously explained by Abunasef et al. [75], who mentioned that STZ caused severe degenerative changes by reducing the size and number, especially in the center of the islets. The section of the pancreas of the FCM treated group (Figure 1G3) showed mild deteriorations in the islets of Langerhans that were distinctly increased in size, and the severity of degenerative and necrotic changes in the islet cells of Langerhans was less than those in G2 (Figure 1 G2(a,b)). The importance of restoring the activity of islets of Langerhans in the treatment of DM was previously explained [76] as targeting the pancreatic β-cells is considered one of the most promising strategies for treating diabetes [8]. Their results showed that the administration of camel milk caused the restoration of insulin secretion in diabetic rats, which means that the Langerhans islets β-cells restored their activity. Moreover, camel milk has antitoxic effects that reduce the dangerous effect of STZ and contains insulin-like substances, especially when fermented with probiotic strains that help modulate glucose levels [7,15,17]. Furthermore, the probiotic bacteria used in fermentation may be the reason behind producing bioactive peptides with an antidiabetic activity [77]. The positive effects of *S. officinalis* hydroalcoholic extract on STZ-diabetic rats were also demonstrated (Figure 1G4). Mild atrophy in islets of Langerhans and interlobular fibrosis and no necrobiosis in acini were observed. The explanation of Mahdizadeh et al. [64] goes hand in hand with our results presented in Table 2 regarding the effect of *S. officinalis* administered to STZ-diabetic rats. They demonstrated that *S. officinalis* extract is rich in phenolic and flavonoid compounds, such as rosmarinic acid. They concluded that *S. officinalis* extract inhibits DNA damage, reduces lipid peroxidation, protects neural cells against H_2_O_2_, has hypoglycemic effects, and may be used to treat various types of diabetes. The recovery in the pancreatic structure for rats in G5, G6, and G7 was markedly appeared (Figure 1G5–G7), especially for G6, as a result of the combination of FCM and SOHE at 50 mg kg^−1^, which mean that this combination is highly recommended for treatment of DM.

## 5. Conclusions

The antidiabetic potential of probiotic camel milk or *S. officinalis* separately was studied and confirmed in the present study. The current study innovatively investigated the antioxidative and antidiabetic potential of FCM combined with SOHE in the form of functional beverages. It could be concluded that the *S. officinalis* is rich in various phenolic compounds, especially flavonoids with a superior antioxidant capacity. Quantification of phenolics obviously indicated that *S. officinalis* contained valuable amounts of Flavonoids which support its functional and therapeutical properties. FCM combined with *S. officinalis* protects rats against diabetes complications and oxidative stress, as evidenced in our study. The protective efficacy might arise from the synergistic effect of FCM and *S. officinalis* which can modulate glucose levels and attenuate diabetes complications. This superior activity has been confirmed using biochemical and histopathological examinations. Therefore, obtained findings could help to explain the therapeutical efficacy of innovative FCM incorporating SOEH formulated in the current study. It encouraged us to recommend that combining *S. officinalis* with FCM is beneficial and profitable for controlling diabetes mellitus.

## Figures and Tables

**Table 1:** Total phenolic content, total carotenoids, total flavonoids, total flavonols, and relative potential antioxidant activities of *S. officinalis* leaves (mean ± SE), *n* = 6.

Item	*S. officinalis*
TPC (mg GAE g^−1^)	102.81 ± 2.14
TC (µg g^−1^)	4.11 ± 0.73
TF (mg QE g^−1^)	37.57 ± 1.98
TFL (mg QE g^−1^)	53.87 ± 0.91
DPPH (µmol of TE g^−1^)	337.62 ± 4.15
ABTS (µmol of TE g^−1^)	374.31 ± 3.48
β-CB * (RAA) %	63.27 ± 3.25
CA (mg g^−1^)	71.21 ± 3.17

*: relatively calculated based on BHA as 100%, RAA: relative antioxidant activity.

**Table 2:** Quantitative analysis of phenolic compounds in *S. officinalis* leaves (mean ± SE), *n* = 3.

Item	No.	Compound	SOHE (mg 100 g^−1^)
Phenolic acids	1	Pyrogallol	-
	2	Quinol	-
	3	3-Hydroxytyrosol catechol	-
	4	*p*-Hydroxy benzoic acid	23.28 ± 1.25
	5	Caffeic acid	39.15 ± 2.17
	6	Chlorogenic acid	1.27 ± 0.21
	7	Cinnamic acid	28.34 ± 3.27
	8	Ellagic acid	5.57 ± 0.78
	9	Vanillic acid	49.73 ± 4.58
	10	Ferulic acid	814.17 ± 14.87
	11	Gallic acid	1.17 ± 0.14
	12	*O*-coumaric acid	7.08 ± 0.87
	13	*p*-coumaric acid	12.27 ± 1.59
	14	Benzoic acid	89.37 ± 5.47
	15	Rosmarinic acid	13.35 ± 2.14
	16	Syringic acid	5.09 ± 1.85
Flavonoids	1	Catechin	0.25 ± 0.03
	2	Epicatechin	98.12 ± 4.17
	3	Kaempferol	356.52 ± 12.01
	4	Myricetin	18.81 ± 3.27
	5	Naringin	18.18 ± 2.71
	6	Hispidulin	13.17 ± 2.07
	7	Cirsimaritin	89.43 ± 5.13
	8	Quercetin	91.07 ± 2.64
	9	Luteolin-7-O-glucoside	25.18 ± 3.27
	10	Luteolin	87.12 ± 1.78
	11	Rutin	19.67 ± 3.17
	12	apigenin	97.17 ± 1.89
	13	Chrysin	102.57 ± 3.18
	14	Resveratrol	1876.95 ± 23.49

SOHE: *S. officinalis* hydroalcoholic extraction, phenolic acids were identified at 28 nm, and flavonoids were identified at 365 nm, -: Not detected.

**Table 3:** Effect of probiotic-enriched fermented camel milk (FCM) combined with *S. officinalis* on weight gain, RBG, and FBG in STZ-induced diabetes in rats.

Groups	Weight Gain %		RBG			FBG
	Weak-2	Weak-4	Weak-0	Weak-2	Weak-4	
G1	29.59 ± 1.68 ^a^	42.60 ± 0.48 ^a^	100.17 ± 2.23 ^d^	104.67 ± 1.87 ^e^	104.67 ± 1.83 ^g^	73.07 ± 4.70 ^d^
G2	1.72 ± 0.07 ^d^	2.58 ± 0.16 ^d^	341.50 ± 17.83 ^bc^	399.33 ± 8.17 ^a^	291.67 ± 16.84 ^a^	201.74 ± 10.93 ^a^
G3	17.16 ± 1.02 ^b^	27.25 ± 1.43 ^b^	358.17 ± 14.26 ^ab^	316.67 ± 17.26 ^b^	264.00 ± 5.45 ^b^	130.25 ± 9.70 ^b^
G4	11.66 ± 4.82 ^c^	15.62 ± 1.07 ^c^	344.50 ± 13.45 ^b^	281.17 ± 11.12 ^c^	187.67 ± 11.44 ^c^	113.35 ± 37.13 ^bc^
G5	12.36 ± 1.34 ^c^	17.92 ± 1.17 ^c^	378.67 ± 12.95 ^a^	289.83 ± 14.73 ^cb^	168.17 ± 4.32 ^d^	107.50 ± 14.41 ^bc^
G6	29.36 ± 2.69 ^a^	29.18 ± 1.14 ^b^	357.83 ± 23.98 ^ab^	208.67 ± 24.37 ^d^	133.00 ± 4.50 ^f^	103.82 ± 7.41 ^bc^
G7	23.68 ± 3.57 ^a^	26.32 ± 2.34 ^b^	320.33 ± 16.99 ^bc^	174.83 ± 13.98 ^d^	159.33 ± 3.09 ^e^	101.09 ± 6.17 ^bc^

G1–G7: Experimental groups see materials and methods; Section 2.6, RBG: Random blood glucose, FBG: Fasting blood glucose level measured in blood serum of 12-h fasted rats, ^a, b, c, d, e, f^ and ^g^: There is no significant difference (*p* > 0.05) between any two means, within the same column have the same superscripted letters.

**Table 4:** Effect of probiotic-enriched FCM combined with *S. officinalis* on lipid profile and Atherogenic index in STZ-induced diabetes in rats.

Groups	Lipid Profile Parameters					
	TG	CHO	HDL-CHO	LDL-CHO	VLDL-CHO	AI
G1	50.32 ± 3.12 ^b^	106.22 ± 8.87 ^b^	42.39 ± 4.64 ^b^	53.76 ± 3.10 ^bc^	10.06 ± 0.88 ^c^	0.10 ± 0.21 ^b^
G2	97.92 ± 4.86 ^a^	176.36 ± 14.95 ^a^	32.61 ± 3.23 ^d^	124.16 ± 6.59 ^a^	19.58 ± 1.37 ^a^	0.50 ± 0.18 ^a^
G3	62.70 ± 4.59 ^b^	119.87 ± 7.17 ^b^	39.13 ± 3.07 ^bc^	68.20 ± 8.29 ^b^	12.54 ± 1.30 ^b^	0.21 ± 0.14 ^b^
G4	57.84 ± 3.57 ^b^	108.22 ± 10.87 ^b^	43.48 ± 5.81 ^b^	53.18 ± 9.99 ^bc^	11.57 ± 1.01 ^b^	0.22 ± 0.32 ^b^
G5	49.38 ± 6.10 ^b^	108.97 ± 5.00 ^b^	45.65 ± 3.02 ^b^	53.44 ± 3.39 ^bc^	9.87 ± 1.73 ^bc^	0.01 ± 0.29 ^c^
G6	47.56 ± 4.59 ^b^	98.87 ± 5.52 ^b^	55.43 ± 5.74 ^a^	34.13 ± 4.51 ^c^	9.31 ± 1.48 ^bc^	−0.07 ± 0.21 ^d^
G7	55.33 ± 5.48 ^b^	113.56 ± 5.32 ^b^	38.04 ± 4.10 ^c^	64.45 ± 4.11 ^b^	11.07 ± 1.55 ^b^	0.16 ± 0.31 ^b^

G1–G7: Experimental groups see materials and methods; Section 2.6, TG: Triglycerides, CHO: Total cholesterols, HDL-CHO: High-density lipoprotein-cholesterols, LDL-CHO: Low-density lipoprotein-cholesterols, VLDL-CHO: Very low-density lipoprotein-cholesterols, AI: Atherogenic index, ^a, b, c^ and ^d^: There is no significant difference (*p* > 0.05) between any two means within the same column with the same superscripted letters.

**Table 5:** Effect of probiotic-enriched FCM combined with *S. officinalis* on liver functions in STZ-induced diabetes in rats.

Groups	Liver’s Functions				
	ALT (U L^−1^)	AST(U L^−1^)	ALP(U L^−1^)	T. Bili (mg dL^−1^)	D. Bili (mg dL^−1^)
G1	42.99 ± 1.98 ^bc^	95.53 ± 5.03 ^c^	74.61 ± 3.93 ^b^	0.85 ± 0.01 ^c^	0.23 ± 0.04 ^b^
G2	66.53 ± 4.94 ^a^	133.37 ± 5.31 ^a^	100.03 ± 3.01 ^a^	1.44 ± 0.08 ^a^	0.36 ± 0.08 ^a^
G3	51.40 ± 1.01 ^b^	108.99 ± 8.14 ^b^	85.12 ± 6.36 ^b^	0.88 ± 0.01 ^c^	0.26 ± 0.05 ^ab^
G4	47.13 ± 3.12 ^b^	102.81 ± 4.10 ^bc^	73.75 ± 1.91 ^c^	1.07 ± 0.12 ^bc^	0.21 ± 0.03 ^b^
G5	45.48 ± 2.61 ^bc^	94.65 ± 5.28 ^c^	74.44 ± 1.75 ^c^	1.14 ± 0.05 ^b^	0.24 ± 0.04 ^b^
G6	39.69 ± 4.15 ^c^	92.45 ± 6.51 ^c^	69.82 ± 2.40 ^c^	1.14 ± 0.05 ^b^	0.23 ± 0.04 ^b^
G7	43.83 ± 2.94 ^bc^	101.14 ± 2.95 ^bc^	78.99 ± 2.31 ^b^	0.92 ± 0.08 ^c^	0.26 ± 0.04 ^ab^

^a, b^ and ^c^: No significant difference (*p* > 0.05) between any two means within the same column have the same superscripted letters. ALT: Alanine aminotransferase, AST: Aspartate aminotransferase, ALP: Alkaline phosphatase, T. Bili: Total bilirubin, D. Bili: Direct bilirubin.

**Table 6:** Effect of probiotic-enriched FCM combined with *S. officinalis* on kidneys’ functions in STZ-induced diabetes in rats.

Group	Kidneys’ functions					
	T. Protein (g dL^−1^)	Albumin (g dL^−1^)	Globulin (g dL^−1^)	Createnine (mg dL^−1^)	Urea (mg dL^−1^)	BUN (mg dL^−1^)
G1	8.51 ± 0.42 ^ab^	3.87 ± 0.26 ^ab^	4.64 ± 0.57 ^a^	0.69 ± 0.08 ^b^	50.68 ± 2.27 ^d^	19.68 ± 1.34 ^bc^
G2	6.25 ± 0.19 ^d^	2.85 ± 0.19 ^c^	3.41 ± 0.31 ^b^	1.55 ± 0.09 ^a^	97.56 ± 3.48 ^a^	27.76 ± 1.63 ^a^
G3	7.03 ± 0.18 ^c^	3.25 ± 0.30 ^bc^	3.78 ± 0.32 ^ab^	0.85 ± 0.05 ^b^	65.34 ± 5.39 ^b^	22.07 ± 2.02 ^bc^
G4	7.36 ± 0.23 ^bc^	3.31 ± 0.20 ^bc^	4.05 ± 0.22 ^a^	0.79 ± 0.05 ^bc^	63.53 ± 3.18 ^b^	22.43 ± 1.34 ^bc^
G5	8.03 ± 0.24 ^abc^	3.48 ± 0.14 ^abc^	4.55 ± 0.34 ^a^	0.72 ± 0.04 ^c^	56.96 ± 1.96 ^c^	21.12 ± 1.25 ^bc^
G6	8.70 ± 0.25 ^a^	4.28 ± 0.23 ^a^	4.41 ± 0.45 ^a^	0.69 ± 0.05 ^c^	51.04 ± 3.01 ^cd^	21.26 ± 1.13 ^bc^
G7	7.54 ± 0.34 ^abc^	3.06 ± 0.19 ^bc^	4.48 ± 0.29 ^a^	0.77 ± 0.05 ^bc^	62.35 ± 6.95 ^b^	20.77 ± 1.28 ^bc^

G1–G7: Experimental groups see materials and methods; Section 2.6, BUN: Blood urea nitrogen, ^a, b, c^ and ^d^: No significant difference (*p* > 0.05) between any two means within the same column with the same superscripted letters.

**Table 7:** Effects of oral administration of probiotic-enriched FCM combined with *S. officinalis* on antioxidant biomarkers in Streptozotocin-induced diabetes in rats (mean ± SE), *n* = 6.

Group	Antioxidant Biomarkers			
	GSH (µg dL^−1^)	MDA (n mol mL^−1^)	CAT (U L^−1^)	SOD (U L^−1^)
G1	62.51 ± 4.38 ^ab^	21.25 ± 1.33 ^c^	56.96 ± 5.64 ^bc^	83.63 ± 1.85 ^b^
G2	44.91 ± 2.66 ^d^	36.43 ± 4.37 ^a^	39.53 ± 2.92 ^d^	56.14 ± 0.51 ^e^
G3	50.40 ± 1.37 ^c^	23.57 ± 1.85 ^c^	50.21 ± 4.59 ^c^	70.17 ± 0.64 ^d^
G4	49.88 ± 4.80 ^c^	19.65 ± 3.59 ^c^	64.92 ± 4.30 ^ab^	79.31 ± 0.84 ^c^
G5	65.13 ± 4.48 ^ab^	16.14 ± 1.98 ^d^	65.65 ± 5.86 ^ab^	84.79 ± 0.74 ^b^
G6	69.06 ± 5.08 ^a^	15.05 ± 2.78 ^d^	75.08 ± 5.45 ^a^	91.54 ± 0.79 ^a^
G7	50.96 ± 7.45 ^c^	27.32 ± 2.74 ^b^	56.88 ± 6.22 ^bc^	70.17 ± 0.46 ^d^

G1–G7: Experimental groups see materials and methods; Section 2.6, GSH: Reduced glutathione, MDA: Malonaldehyde, CAT: Catalase, SOD: Superoxide dismutase, ^a, b, c, d^ and ^e^: No significant difference (*p* > 0.05) between any two means within the same column with the same superscripted letters.

**Table 8:** The severity of histopathological alteration in rat pancreas underlying structure of different experimental groups treated by probiotic-enriched FCM combined with *S. officinalis*.

	G1	G2	G3	G4	G5	G6	G7
Atrophy in islets of Langerhans	–	+++	–	+	+	–	–
Necrobiosis in acini	–	++	+	–	–	–	–
Interlobular fibrosis	–	+++	+	+	–	–	–

G1–G7: Experimental groups see materials and methods; Section 2.6, +++ = severe, ++ = moderate, + = mild, – = nil.

## Data Availability

The data are contained within the article.

## Abbreviations

ABTS: 2:2′-azino-bis(3-ethylbenzothiazoline-6-sulfonic acid); ALT, Alanine aminotransferase; AOA, Antioxidant activity; AST, Aspartate aminotransferase; BHA: Butylated hydroxyanisole; CAT, Catalase; CHO, Total cholesterol; DPPH: 1,1-diphenyl-2-picryl hydrazine; dw: Dry weight; FBG, Fasting blood glucose; FCM, Fermented camel milk; GA: Gallic acid; GAE: Gallic acid equivalent; GSH: Reduced-glutathione; HDL, High-density lipoproteins; LDL, Low-density lipoproteins; MDA: Malonaldehyde; QE: Quercetin equivalent; RAA: Relative antioxidant activity; RBG: Random blood glucose; RSA: Radical scavenging activity; SE: Standard error; SOD: Superoxide dismutase; SOHE: *S. officinalis* hydroalcoholic extract; TBA, Thiobarbituric acid; TC: Total carotenoids; TE: Trolox equivalents; TF: Total flavonoids; TFL, Total flavonols; TG, Triglycerides; TPC, Total phenolic compounds; VLDL, Very Low-density lipoproteins.
